# Interactive Somatosensory Games in Rehabilitation Training for Older Adults With Mild Cognitive Impairment: Usability Study

**DOI:** 10.2196/38465

**Published:** 2022-07-14

**Authors:** Chien-Hsiang Chang, Chung-Hsing Yeh, Chien-Cheng Chang, Yang-Cheng Lin

**Affiliations:** 1 Department of Industrial Design National Cheng Kung University Tainan City Taiwan; 2 Faculty of Information Technology Monash University Melbourne Australia; 3 Department of Industrial Design National United University Miaoli Taiwan

**Keywords:** dementia, elderly, usability, gesture recognition, card recognition rehabilitation, interactive somatosensory game

## Abstract

**Background:**

In aging societies, dementia risk increases with advancing age, increasing the incidence of dementia-related degenerative diseases and other complications, especially fall risk. Dementia also escalates the care burden, impacting patients, their families, social welfare institutions, and the social structure and medical system.

**Objective:**

In elderly dementia, traditional card recognition rehabilitation (TCRR) does not effectively increase one’s autonomy. Therefore, from the usability perspective, we used the Tetris game as a reference to develop an interactive somatosensory game rehabilitation (ISGR) with nostalgic style for elders with mild cognitive impairment (MCI). Through intuitive gesture-controlled interactive games, we evaluated subjective feelings concerning somatosensory game integration into rehabilitation to explore whether the ISGR could improve the willingness to use and motivation for rehabilitation among elders with MCI.

**Methods:**

A total of 15 elders with MCI (7 males and 8 females with an average age of 78.4 years) underwent 2 experiments for 15 minutes. During experiment 1, TCRR was performed, followed by completing the questionnaire of the System Usability Scale (SUS). After 3-5 minutes, the second experiment (the ISGR) was conducted, followed by completing another SUS. We used SUS to explore differences in impacts of TCRR and ISGR on willingness to use among elders with MCI. In addition, we further investigated whether the factor of gender or prior rehabilitation experience would affect the rehabilitation willingness or not.

**Results:**

The novel ISGR made the elderly feel interested and improved their willingness for continuous rehabilitation. According to the overall SUS score, the ISGR had better overall usability performance (73.7) than the TCRR (58.0) (*t*_28_=–4.62, *P*<.001). Furthermore, the ISGR individual item scores of “Willingness to Use” (*t*_28_=–8.27, *P*<.001), “Easy to Use” (*t*_28_=–3.17, *P*<.001), “System Integration” (*t*_28_=–5.07, *P*<.001), and “Easy to Learn” (*t*_28_=–2.81, *P*<.001) were better than TCRR. The somatosensory game was easier to learn and master for females than for males (*t*_13_=2.71, *P*=.02). Besides, the ISGR was easier to use (*t*_12_=–2.50, *P*=.02) and learn (*t*_14_=–3.33, *P*<.001) for those without prior rehabilitation experience. The result indicates that for elders with no rehabilitation experience ISGR was easier to use and simpler to learn than TCRR.

**Conclusions:**

Regardless of prior rehabilitation experience, the ISGR developed in this study was easy to learn and effective in continuously improving willingness to use. Furthermore, the adoption of a nostalgic game design style served the function of cognitive training and escalated interest in rehabilitation. The ISGR also improved user stickiness by introducing different game scenarios and difficulties, increasing long-term interest and motivation for rehabilitation. For future research on the adoption of interactive somatosensory games in rehabilitation, additional rehabilitation movements can be developed to benefit the elderly with MCI.

## Introduction

### Background

Owing to social changes and medical advancement, the global demographic structure is becoming an aging society. The World Health Organization (WHO) [1] has reported that the number of deaths caused by dementia has doubled in recent years, making it the fifth leading cause of death worldwide. At present, there are approximately 50 million people with dementia; besides, the prevalence of dementia among the elderly over 60 years old is 5%-8%. This number is expected to reach 152 million by 2050. Data suggest that the incidence of dementia is increasing annually, which has prompted the WHO to launch the “Global action plan on the public health response to dementia” with reduction in the risk of dementia as one of the priorities. As the size of the local population with dementia rapidly elevates, the Taiwanese government has initiated a new version of the long-term care plan, the “Long-term Care 2.0 in Taiwan,” which prioritizes the care of patients with dementia and the establishment of a friendly dementia care model [2]. Song and Wang [3] have indicated that while there are no existing cures for dementia, rehabilitation therapy can be used as a way of daily training. Rehabilitation training, which can be divided into functional, psychological, behavior management, and other activities, such as hand exercises and creative and mind-stimulating activities that promote community communication, is capable of effectively improving the quality of life of patients [4]. Similarly, Fried et al [5] have reported that finger exercise can not only effectively prevent dementia, but also alleviate anxiety as well as enhancing cold hands and feet, stabilizing blood pressure, and improving sleep quality.

Among various treatment methods for dementia, traditional rehabilitation such as card recognition training utilizes commercially available playing cards for cognitive training. However, its long-term use can neither enhance the autonomy of the elderly nor increase user stickiness. On the contrary, digital games have now been introduced as an emerging cognitive training mode and are referred to as “serious games” [6]. Unlike general entertainment games, serious games are called “serious” because they “replace serious themes with interesting games, which are purposeful and helpful to players” [6-9]. With the maturity and popularization of digital technology, increasing attention has been drawn to the application of interactive games in training, believing that it is an effective training tool [10-12]. In terms of game-based training, Zarit et al [13] proposed a combination of training (rehabilitation) and games, which could not only realize the training effect through games, but also improve users’ motivation and willingness to use via high interactivity and objectivity.

This study found that existing card-based rehabilitation not only failed to promote the autonomous use among the elderly, but also triggered hand movements only 3-5 times per minute. Therefore, this study developed a nostalgic game (Tetris) combined with gesture detection technology, allowing elders use their finger movements to control the interactive game. Concurrently, the nostalgic game stimulated the elder’s memory (returned to their childhood days) as well as increased their frequency of finger movement (8-10 times per minute), thereby effectively improving user motivation as well as achieving a cognitive training effect. However, for the elderly, the operation of interactive somatosensory game rehabilitation (ISGR) was more complicated than that of the traditional card recognition rehabilitation (TCRR). Therefore, whether the operation of ISGR would compromise the willingness to use for elders’ rehabilitation remains an unsolved question. Would the factors such as prior rehabilitation experience and gender be affected by their rehabilitation motivation? To answer these questions, the System Usability Scale (SUS) [14] was adopted to investigate the subjective feelings toward the ISGR and willingness to use by elders with mild cognitive impairment [MCI], with the results also compared with the TCRR.

The SUS was developed by Brooke [14] to explore the system-related experience of users, whose feedback can be used as a basis for understanding/improving the usability of the system. Its main purpose is to detect the subjective feelings of users after operating the system and to evaluate and ensure the usability of the system. The SUS collects users’ subjective evaluations of products (or systems) by comparing their performances in different tasks and identifies usability and user satisfaction levels, thereby allowing developers to quickly determine the usability of the product (or system) [15]. In addition, according to relevant medical literature, Yen and Bakken [16] proposed the system development life cycle to evaluate the representative expected value and usability for each different development stage [17,18].

This study aimed to develop an interactive somatosensory game to promote the rehabilitation motivation of elders with MCI, to ensure they were satisfied with the ISGR and could continuously use the ISGR. Sections of the paper included discussions of relevant literature on dementia, the design process of the interactive somatosensory game, usability experiments, and analysis and discussion on the SUS.

### Literature Review

#### Overview

In this section, a literature review and analysis of the importance of dementia rehabilitation, the application of gesture recognition to dementia treatment, and the efficacy of nostalgia therapy on cognitive impairment were conducted. Furthermore, this section will discuss the development status of the application of gesture-based interactive somatosensory games in the rehabilitation.

#### Importance of Dementia Rehabilitation

The cognitive function includes emotion, personality, perception, memory, abstract judgment, and the ability to express things, and MCI refers to the transitional stage during which a normal person develops dementia (increasing with advancing age) [19]. During MCI, people’s daily life is basically unimpacted, although their decline in memory exceeds the normal range. While there is currently no standard for the diagnosis of MCI, Petersen et al [20] proposed that individuals should be diagnosed with MCI if they experienced memory impairment and yet had a normal daily life, normal overall cognitive function, normal memory for their age, and no signs of dementia. Studies indicate that about 10%-15% of the patients with MCI could progress to dementia without receiving proper treatment [19].

Despite being a disease, dementia is thought of by many family members as a common aging phenomenon and the importance of its treatment is therefore often neglected. Once the severity of dementia and cognitive impairment elevates, the difficulty of care increases correspondingly. Furthermore, as the patient ages, the risk of fall also rises, with a study reporting that the risk of fall of elderly people with dementia was substantially higher than that of those without [21]. While approximately 20%-30% of the elderly over the age of 65 have experienced falls, that rate is twice as high among the elderly with dementia [22]. Falls not only injure the elderly, but also bring them into a vicious cycle of functional deterioration. Therefore, to prevent dementia from causing additional complications, it is of particular significance to provide the elderly with appropriate cognitive rehabilitation training to improve the cognitive function and delay the deterioration of dementia. Research indicated that cognitive training and rehabilitation methods exerted a positive effect on patients with dementia with cognitive decline. As a nondrug treatment method, rehabilitation not only reduces burdens on the National Health Insurance, but also improves the quality of life and reduces the impact of mental illness on the elderly [23]. Therefore, developing health care for the elderly with dementia as well as delaying and improving the onset of dementia has become a significant social issue that requires immediate attention.

#### Application of Gesture Recognition in the Treatment of Dementia

The incidence of dementia has been on the rise in recent years, making it a common disease among the elderly. Furthermore, its prevalence increases with advancing age [19]. However, rather than a normal aging phenomenon, dementia is a degenerative disease that is yet to be cured by drug treatment. Despite so, the incidence of dementia can be effectively prevented by activities. Regular exercise has been proven effective in preventing and improving dementia in the elderly [24]. This is because exercise can enhance the function of the cerebral cortex, thereby benefiting brain intelligence. In addition, an appropriate amount of exercise can alleviate mental anxiety, which in turn protects neurons [25,26].

Recent studies have verified the correlation between the fingers and the brain. Fried et al [5] reported a direct relationship between the hands and brain activities. He suggested that while the frontal and temporal cortices in the brain supported human’s motor behavior, continuous exercise could affect parietal and frontal regions of the brain that were related to the medial and lateral surface cortices, thereby productively preventing dementia [27]. In addition, Pellegrino and Làdavas [28] indicated that as the control center of various parts of the body, the brain functions through extensive multisensory interactions within a set of interconnected parietal and frontal regions. Taking finger movements as an example, Gardini et al [29] proposed that motor thinking was controlled by a cortical network that mainly involved abstract thinking, cognition, motor control, semantics, and visual image processes.

**Figure 1:**
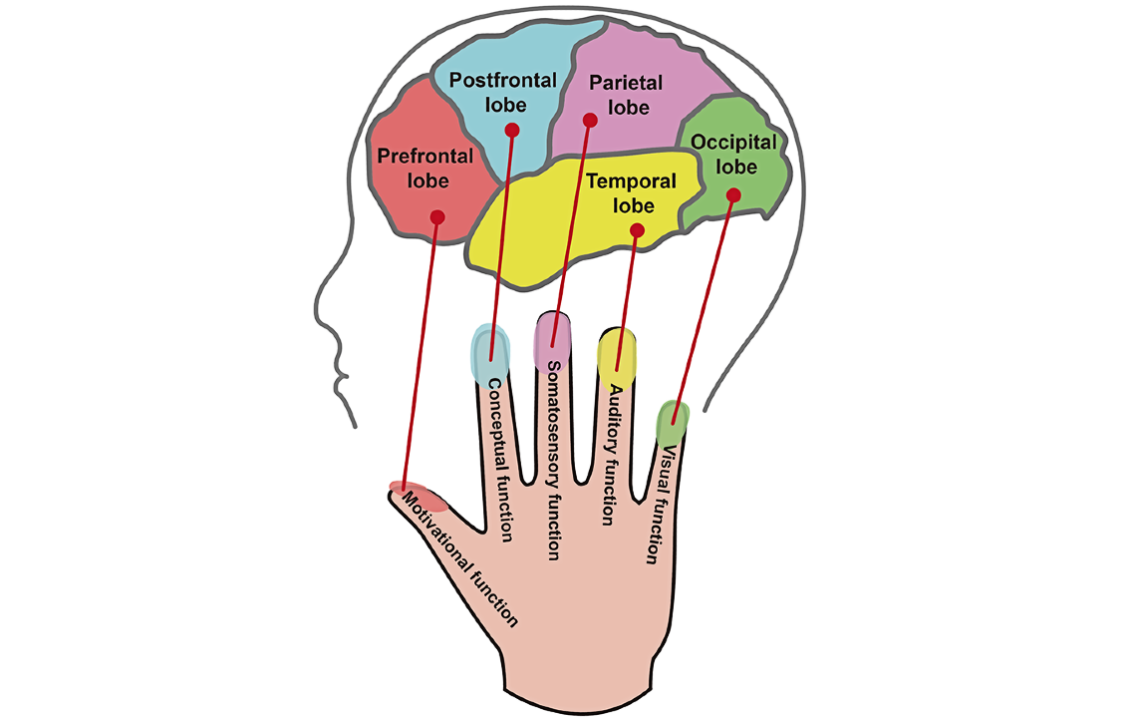
Areas of the hands linked to different brain functions.

The book *Fascinating Dermatoglyphics* by Chen [30] mentioned that the left hand corresponds to the right brain, while the right hand corresponds to the left brain. Each finger had different functions and was linked to different areas of the brain. A brief overview of the book [30] is shown in Figure 1.

- Thumb: Corresponding to the prefrontal lobe, it affects motivational functions (such as communication management, creative leadership, plan execution, introspective will).
- Index finger: Corresponding to the postfrontal lobe, it affects conceptual functions (such as logical reasoning, computational analysis, language expression, spatial concepts).
- Middle finger: Corresponding to the parietal lobe, it affects somatosensory functions (such as somatosensory recognition, orientation judgment).
- Ring finger: Corresponding to the temporal lobe, it affects auditory functions (such as auditory recognition, emotional control, music rhythm, language understanding).
- Little finger: Corresponding to the occipital lobe, it affects visual functions (such as visual recognition, observation and understanding, image observation).

The book *Stimulating the Thumbs to Make the Brain Younger* by Hasegawa [31] mentioned that moving fingers could increase the blood flow in the motor cortex and somatosensory cortex of the brain by more than 10%, making it effective in preventing dementia [31]. Furthermore, stimulating fingers not only reduces the onset of dementia symptoms, but also improves the blood circulation and motor functions of the human body. In the modern society, our brain constantly works in a stressful environment. Although unaware of it, long-term stress can affect our health. By contrast, finger movements can promote the differentiation and maturation of areas of the brain, thereby exhibiting a positive impact on our health.

With the advancement of science and technology, the cost of production is becoming increasingly lower, making technology products more accessible and their implementation in medical care an important development goal. At present, multiple gesture recognition sensors are commercially available, of which controller-based visual recognition technologies, such as leap motion (LM) and Kinect (Microsoft), are the most common tools for hand rehabilitation. The therapeutic value of these technological tools has been well verified in previous studies [32,33]. Smeragliuolo et al [34] utilized LM in clinical trials and confirmed that it was very sensitive in tracking the hand motion and could provide clinically valuable data.

#### Efficacy of Nostalgia Therapy on Cognitive Impairment

Treatment methods of dementia can be divided into drug treatment and nondrug treatment; the latter includes nostalgia therapy, music therapy, serious training, art therapy, among others [35]. The goal is to improve the quality of life of patients and to prevent and delay the incidence of diseases. Although currently no drug treatment can completely prevent or cure dementia-related degenerative diseases, both drug and nondrug methods are capable of delaying the progression of the disease or alleviating symptoms.

Nostalgia therapy uses a set of planned nursing activities to remind the elderly of people, events, and things that have special meanings to them and guide them to tell their experiences, thereby helping them realize that their life is meaningful and valuable and regain old memories [36]. It can improve the patient’s self-esteem and self-confidence as well as alleviating depression. As there are currently limited drugs for dementia and a drug cure is yet to be discovered, nondrug treatment has become the mainstream against dementia. Multiple nondrug treatment methods for dementia are available, among which nostalgia therapy has been shown by many studies to demonstrate positive results on the cognitive function and the quality of life of patients [37]. By satisfying the physical, psychological, and social needs of the elderly, nostalgia therapy can help them realize self-integration and improve their mental status as well as quality of life, thereby not only preventing and reducing the incidence of dementia, but also greatly facilitating the elderly to adapt to their daily life. Therefore, nondrug treatments for dementia have received increasing attention in recent years. Research has confirmed that nondrug therapy can reduce the degree of cognitive degradation and the onset of psychobehavioral symptoms [38]. Furthermore, it can be used to communicate with patients with severe dementia. Therefore, nondrug therapy has been commonly adopted in the care of elderly people and is considered an important measure that maintains the physical and mental health as well as the quality of life of the elderly [38].

**Figure 2:**
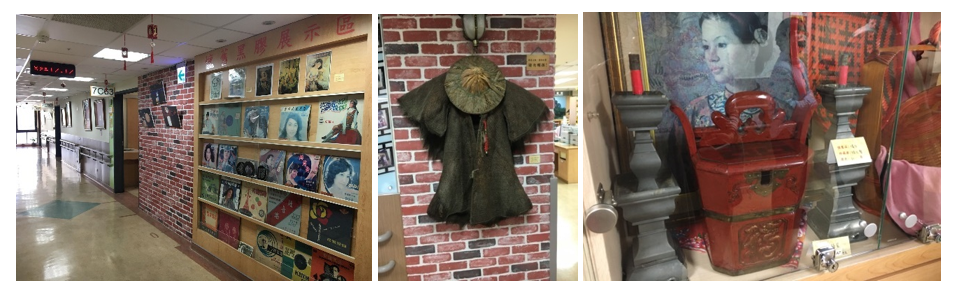
Arrangements of nostalgic objects in the Geriatrics Center of the National Cheng Kung University Hospital.

Autobiographical memory is a very important memory for humans, the loss of which can lead to a disconnection between the past and the present and more severely, the loss of personal identity [39]. Research has shown that nostalgia therapy exhibits a significant impact on the episodic and semantic memory of the autobiographical memory of patients with MCI [40]. In addition, it positively affects the cognition, communication, and emotion of patients with dementia, thereby improving their quality of life, cognitive ability, and social ability [41]. Duru Asiret and Kapucu [42] recruited 62 patients (31 in the experimental group and 31 in the control group) and asked them to participate in a 35-minute therapy course per week for a duration of 12 weeks before getting tested. The results of their experiment confirmed that memory therapy significantly improved cognition and depression in patients with mild or moderate dementia. Similarly, the Geriatrics Center of the National Cheng Kung University Hospital (in Tainan, Taiwan) established a nostalgic space-time walk path in the clinical environment by decorating the path with nostalgic objects. By walking through the path, the dementia symptoms of the elderly were successfully alleviated (as shown in Figure 2).

### Objective

This study aims to explore the integration of interactive somatosensory games into cognitive training and to investigate the feasibility of applying the interactive somatosensory game developed in this study to the rehabilitation of the elderly.

## Methods

### Interactive Somatosensory Game Design

The interactive somatosensory game developed in this study incorporated simple operations and easy-to-learn gameplay, and integrated the gesture recognition technology of LM, which distinguished it from traditional Tetris games. Gesture recognition was used to control the number and the movement of the blocks. This not only made it easier for the elderly to operate, but also facilitated the movement and rehabilitation of their hands through hand exercise, thereby improving the cognitive ability of the elderly and preventing degenerative diseases. The following sections provide an in-depth discussion on the 3 major aspects of the game design, that is, gesture recognition technology, game interface design, and game system design.

### Gesture Recognition Technology

**Figure 3:**
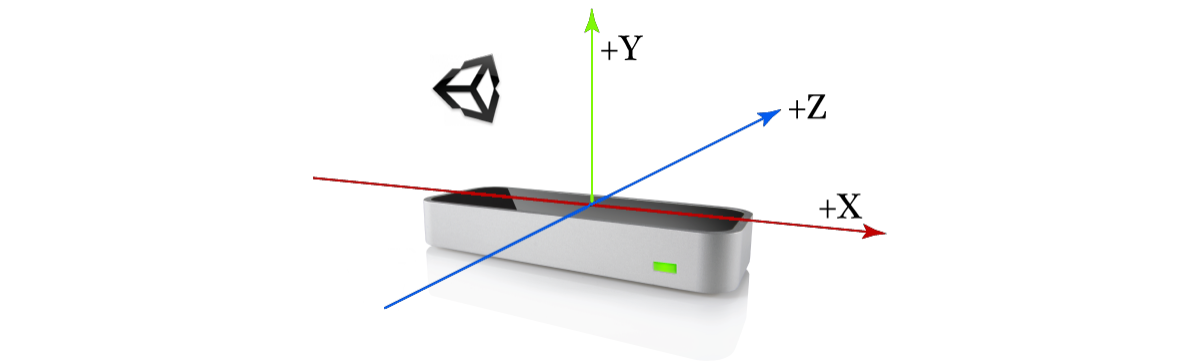
Recognition of hand orientation by the leap motion device.

With the development of science and technology and the rise of human-computer interaction, motion recognition technology has received substantial attention. As a key technology in motion recognition, gesture recognition captures the dynamic gestures of human hands through visual sensor technology. In recent years, as the development cost has decreased and technology has rapidly developed, many dynamic gesture capturing tools have emerged in the market. This study adopted the LM gesture capture tool, which realizes gesture recognition through dual cameras and 3 infrared LEDs (Figure 3). LM can not only recognize multiple hands simultaneously, but also has a submillimeter accuracy and a sensing distance of 25-600 mm above the device [43], making it the most accurate economic gesture recognition tool in the market [44]. The biggest difference between LM and other hand recognition tools such as Microsoft Kinect is that LM is capable of capturing depth data. By calculating the palm center position and other related point positions through the palm orientation and fingertip position, LM can acquire depth information without additional calculations [45]. LM can be utilized in various gesture recognition applications, such as gesture control interface, acquisition of user’s hand skeletons. Therefore, this study adopted LM as the gesture recognition technology. The Unity engine was also utilized to integrate technical tools into game development.

### Game Interface Design

**Figure 4:**
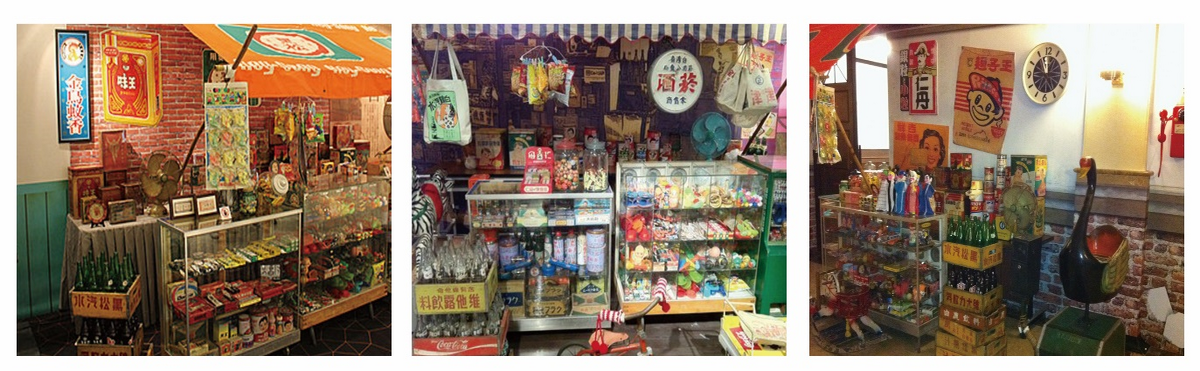
Traditional Taiwanese grocery stores in the 1960s.

**Figure 5:**
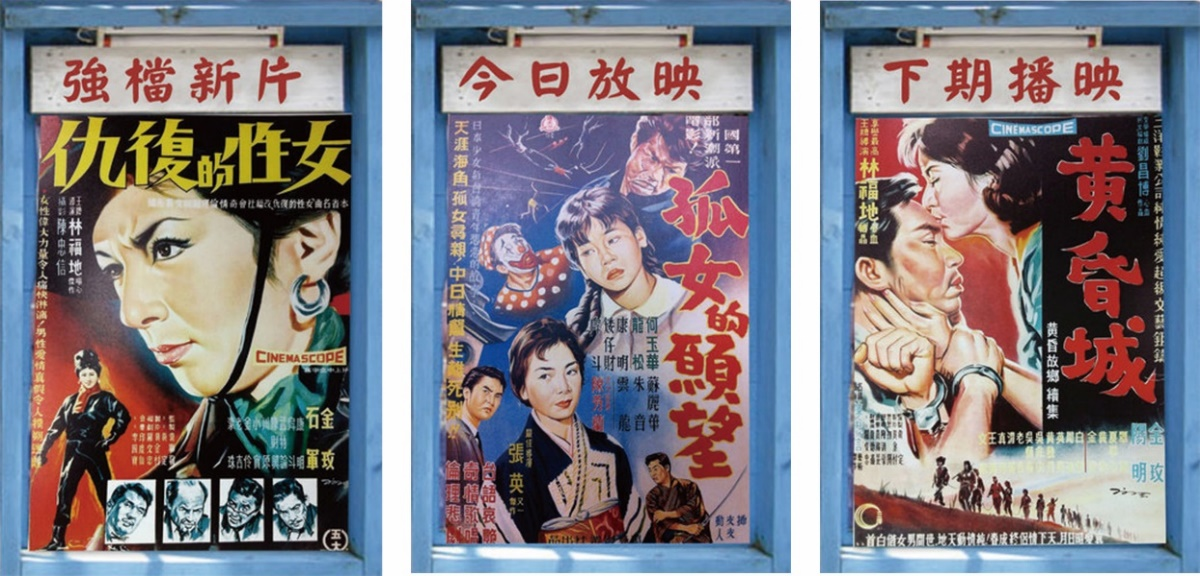
Movie posters in Taiwan in the 1960s designed by Chang.

**Figure 6:**
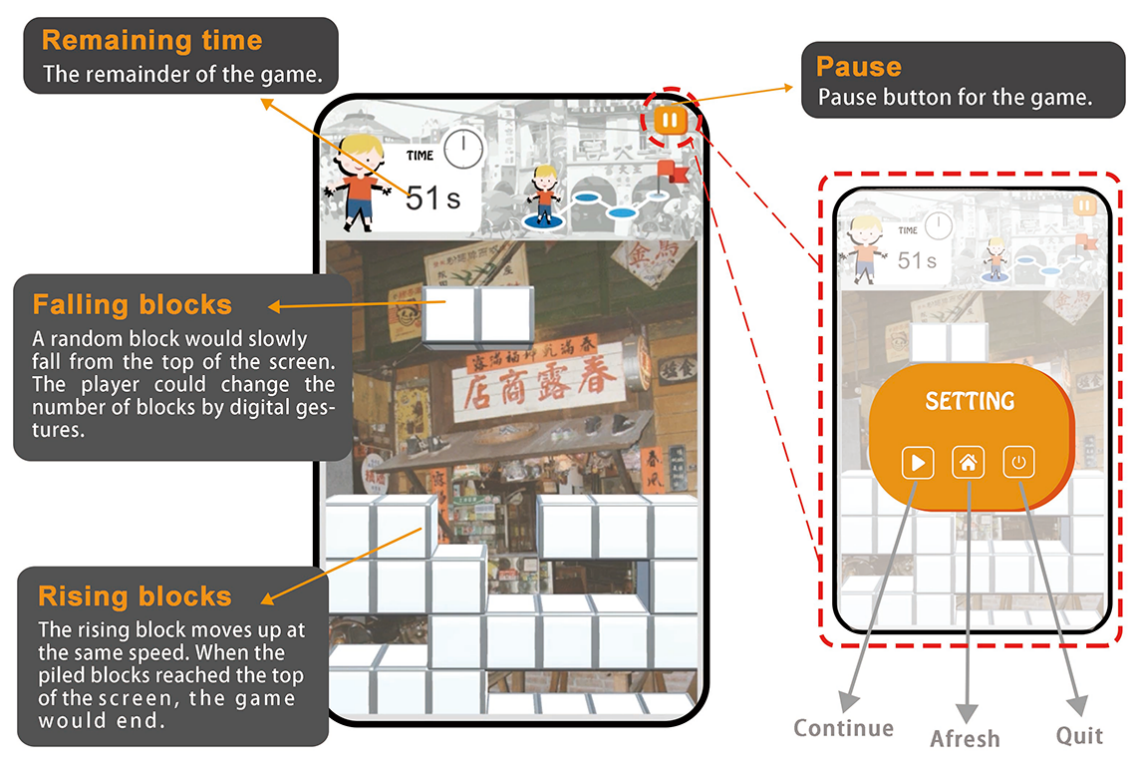
Description of the game interface design.

The game interface design developed in this study was presented in a nostalgic style, providing players with a unique experience through cultural scenes of Taiwan in the 1960s, such as traditional Taiwanese grocery stores, classic movie posters. The background of this game was based on nostalgic designs created by Chang [46], who reinterpreted the nostalgic Taiwanese grocery store scene. Unlike the convenience stores today, the traditional grocery stores in the 1960s sold not only rice, oil, salt, sauce, and tea, but also various colorful dried fruit preserves that filled the shelves of the entire store, making it a common memory of people in the 1960s (Figure 4). Through these traditional grocery stores’ scenes, the elderly (players) felt like they entered a time corridor and were reminded of these beautiful memories during the gameplay. In addition, players were rewarded with classic movie posters after clearing the game, which they could use to share memories with the family and connect with them after the game. The rewards in this study were masterpiece movie posters hand-painted by Chang [46], who drew posters of the most popular movies in Taiwan in the 1960s (Figure 5). By presenting elderly players with the historical atmosphere in the 1960s and letting them walk through the movie scenes and the historical corridor through classic movie posters, the game also connected elderly people’s memory with the story of the movie. By contrast, the remaining time interface of the game was designed by including their children as the countdown image, which symbolized their grandchildren’s company during the gameplay. Therefore, the game improved the relationship between the elderly and his/her families and promoted the former to share and recall the past, thereby delaying the onset of dementia (Figure 6).

### Game System Design

**Figure 7:**
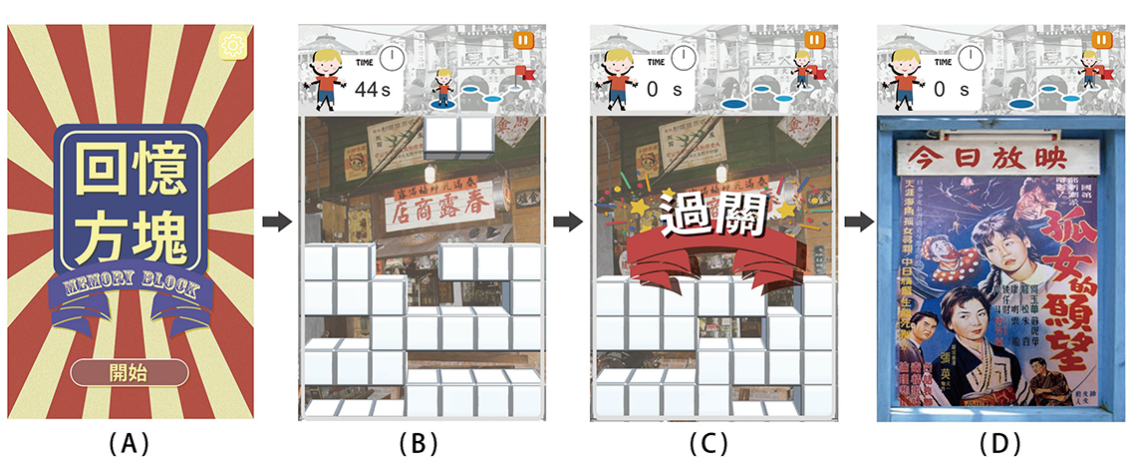
Process of the game developed in this study.

**Figure 8:**
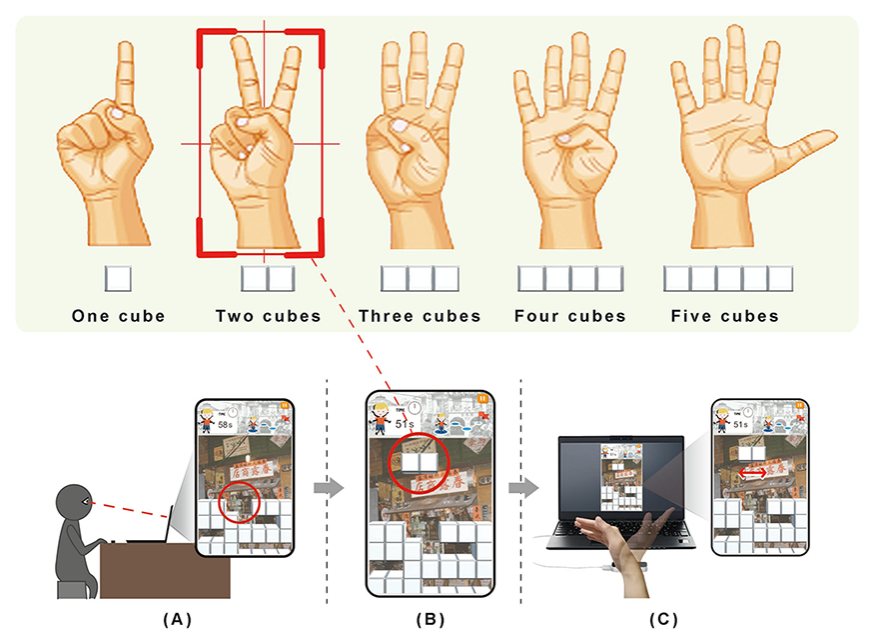
Game operation. (A) Watch the number of the topmost row of blocks when a block falls; (B) change the number of randomly falling blocks through digital gestures; (C) control the movement of the block by swinging the hands left and right.

Once the interactive somatosensory game was initiated, the player was first shown the main screen (Figure 7A) before entering the game (Figure 7B). After the player cleared the level in 1 minute (Figure 7C), a bonus content would be awarded (Figure 7D). At the start of the game, a random block would slowly fall from the top of the screen (Figure 8A). During this process, the player could change the number of blocks by digital gestures (Figure 8B), followed by relocating the falling block to the desired position by moving hands left and right or accelerating the block by moving the hands down (Figure 8C). When the block reached the bottom of the screen or the top of other blocks, it would stop there, while a new random block would appear on top of the screen and start to fall. When a horizontal line was filled with blocks, it would be eliminated, increasing the player’s score. The more lines eliminated simultaneously, the more points the player gets. When the piled blocks reached the top of the screen and could no longer be removed, the game would end.

### Clinical Trial of Interactive Somatosensory Game Rehabilitation

#### Overview

The clinical trial of the experiment was described in this section, including research patients and the experimental design (ie, TCRR and ISGR). The SUS questionnaire with a 5-point scale was adopted to collect quantitative information.

#### Research Patients

**Figure 9:**
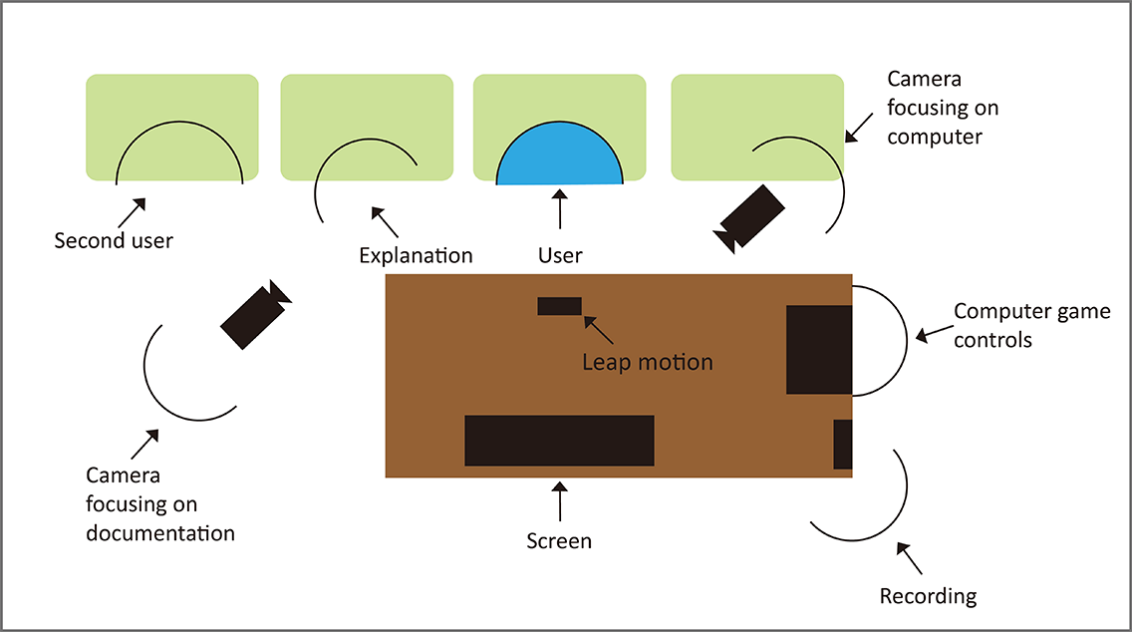
Experimental location.

This study interviewed a total of 15 patients at a Clinic in Tainan, Taiwan, consisting of 7 males (5 with rehabilitation experience and 2 without) and 8 females (2 with rehabilitation experience and 6 without). The average age of the patients was 78.4 years. The inclusion criteria were elderly people over 60 years old who were diagnosed with MCI or were prone to dementia. Participants were also required to be able to cooperate with researchers, follow game instructions, and complete a subjective assessment after rehabilitation. The experimental location of this study is shown in Figure 9.

#### Experimental Design

**Figure 10:**
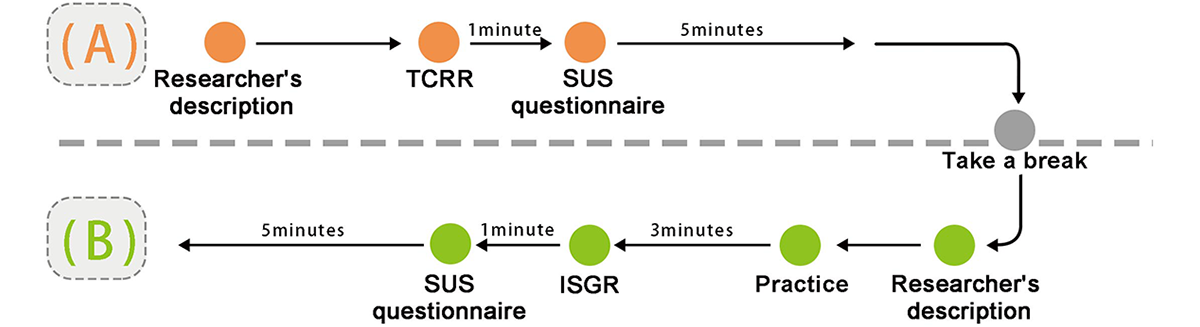
Experiment flow chart. The experiment starts with a (A) TCRR, followed by (B) ISGR. ISGR: interactive somatosensory game rehabilitation; SUS: System Usability Scale; TCRR: traditional card recognition rehabilitation.

The experiment adopted a single-group design, that is, all patients would participate in both TCRR and ISGR. The questionnaire, composed of 10 questions (items), was designed based on the modified SUS [14]. Each question was scored on the 5-point scale (with 1 representing strongly disagree and 5 representing strongly agree) to understand the elderly’s subjective feelings of the system during operation. The purpose of the experiment was to investigate the relationship between the use of TCRR and ISGR among the elderly and to analyze their demands (subjective feelings). The experiment was divided into 2 stages, that is, TCRR and ISGR. Out of 15 patients, 7 (5 males and 2 females) had prior rehabilitation experience, thus having operated on the TCRR before. To make the baseline of previous experience the same for all 15 patients, they all first operated on the TCRR and then on the ISGR. The experiment flowchart is shown in Figure 10.

#### Experiment Stages

##### Experiment of TCRR

**Figure 11:**
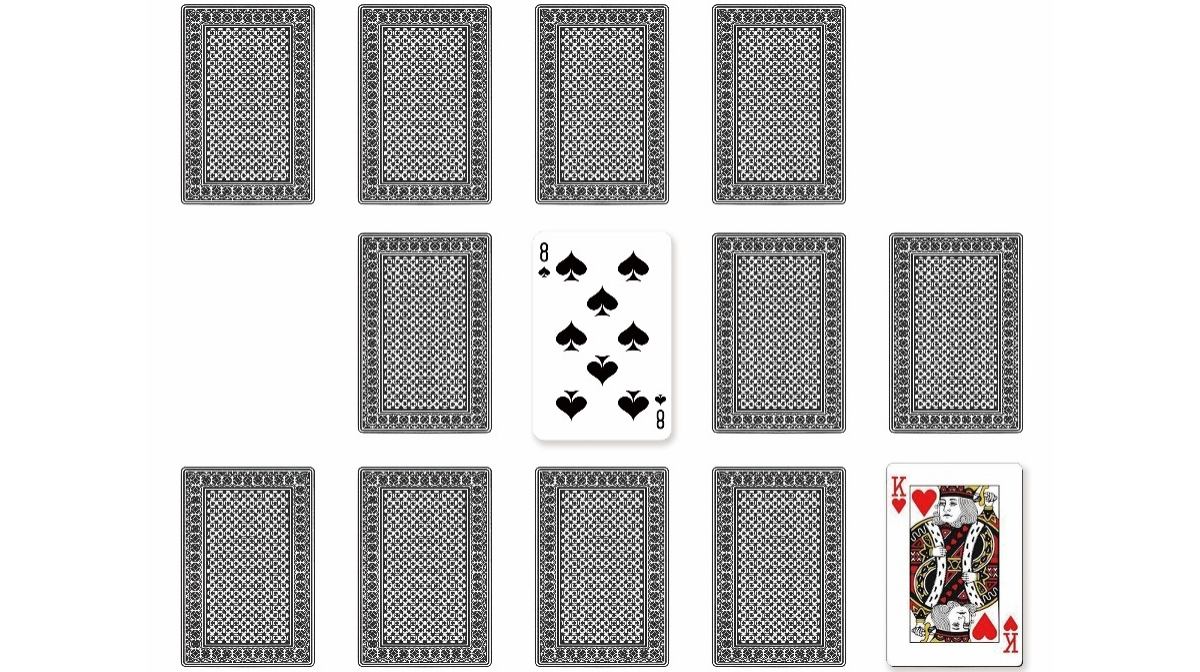
Traditional card recognition rehabilitation.

The TCRR used commercial poker cards for rehabilitation. During the experiment, researchers would cover the cards and place them on the table. The elders could choose any 2 cards and flip them over each time. If the 2 cards were paired (having the same pattern), the elders would gain the opportunity to continue revealing other cards, or otherwise the 2 cards would be flipped back over. The rehabilitation would end when all cards were revealed (Figure 11). In this study, after the operation method was explained by the researcher, the participant would perform the TCRR for 1 minute. Upon completion, the researcher would ask the elders about their subjective feelings of the TCRR using the 5-point scale. The contents of the SUS are listed in Table 1.

**Table 1:** SUS^a^ questionnaire design.

Item	Question description
1	I think that I would like to use this *TCRR*^b^*/ISGR*^c^ frequently.
2	I found the *TCRR/ISGR* unnecessarily complex.
3	I thought the *TCRR/ISGR* was easy to use.
4	I think that I would need the support of a technical person to be able to use this *TCRR/ISGR*.
5	I found the various functions in this *TCRR/ISGR* were well integrated.
6	I thought there was too much inconsistency in this *TCRR/ISGR*.
7	I would imagine that most people would learn to use this *TCRR/ISGR* very quickly.
8	I found the *TCRR/ISGR* very cumbersome to use.
9	I felt very confident using the *TCRR/ISGR*.
10	I needed to learn a lot of things before I could get going with this *TCRR/ISGR*.

^a^SUS: System Usability Scale.

^b^TCRR: traditional card recognition rehabilitation.

^c^ISGR: interactive somatosensory game rehabilitation.

##### Experiment of ISGR

Prior to the formal start of the experiment session, the researcher would first explain the operation of the game verbally, followed by 1 or 2 practices to ensure that the elderly understood the operations correctly. This process would take approximately 3 minutes. During the experiment, the game scenario was randomly selected, and the elderly would use gestures to perform the ISGR. Upon completion, the researcher would ask the elderly about his/her subjective feelings of the ISGR through the 5-point scale. The elderly would verbally answer the questions and the researcher would assist in filling out the records.

### Ethics Approval

This study was approved by the National Cheng Kung University Human Research Ethics Committee (approval number NCKU HREC-F-109-497-2).

## Results

### Overview

The purpose of this study was to investigate whether the ISGR could improve the willingness and motivation for rehabilitation among the elderly with MCI. The study recruited 15 patients, consisting of 7 males and 8 females with an average age of 78.4 years (please see the “Methods” section). Subsequently, the independent (unpaired) sample *t* test was adopted to analyze whether there were any differences in the willingness to use between TCRR and ISGR. This was realized by conducting 2 SUS surveys, with the first enquiring the elderly about their intention to use (willingness to use) the TCRR, and the second enquiring the same group about their intention to use (willingness to use) the ISGR. Table 2 shows the results of the 2 surveys. It should be mentioned that among the 15 patients, 8 (2 males and 6 females) had no rehabilitation experience, whereas the remaining 7 (5 males and 2 females) had prior rehabilitation experience. As Table 2 shows, the overall SUS score for the TCRR is 58.0, while for the ISGR it is 73.7 (*t*_28_=–4.62, *P*<.001). The result shows that the ISGR has better overall usability performance (*average +*) than the TCRR (*poor*) [47,48].

To further investigate, Table 3 shows the individual item benchmarks for the TCRR and the ISGR. For the TCRR, only 2 items (out of 10 items) achieved the *average* benchmark (items 2 and 8) [47,48], thus suggesting that there was a lot of room for improvement in the usability of the TCRR. By contrast, the ISGR has 3 items reaching the *average* level (items 5, 8, and 10) and 5 reaching the *good* level (items 1-3, 7, and 9). Only items 4 and 6 need to improve further. This result indicates that the ISGR presents an average or good experience for elders with MCI when interpreting single items from the SUS [48], as compared with the TCRR. The following analyses examine the effects of prior rehabilitation experience and gender.

**Table 2:** The overall SUS^a^ scores for the TCRR^b^ and ISGR^c^ (n=15 for both).

Patients	Sex	Prior rehabilitation experience: Yes or No	SUS score for TCRR^d^	SUS score for ISGR^e^
1	Male	Yes	62.5	65.0
2	Male	Yes	67.5	82.5
3	Male	No	47.5	77.5
4	Male	No	62.5	70.0
5	Male	Yes	62.5	80.0
6	Male	Yes	60.0	65.0
7	Male	Yes	57.5	75.0
8	Female	Yes	85.0	82.5
9	Female	No	47.5	60.0
10	Female	No	47.5	82.5
11	Female	No	55.0	85.0
12	Female	No	57.5	75.0
13	Female	Yes	60.0	67.5
14	Female	No	50.0	60.0
15	Female	No	47.5	77.5

^a^SUS: System Usability Scale.

^b^TCRR: traditional card recognition rehabilitation.

^c^ISGR: interactive somatosensory game rehabilitation.

^d^Mean (SD) 58.0 (10.01), so rated “poor”.

^e^Mean (SD) 73.7 (8.49), so rated “average +”.

**Table 3:** Basic descriptive statistics and item benchmarks for SUS^a^.

Item	TCRR^b^ (1=lowest, 5=highest)		TCRR score (n=15), mean (SD)	Item benchmark	ISGR^c^ (1=lowest, 5=highest), mean (SD)			ISGR score (n=15), mean (SD)		Item benchmark
	No rehabilitation experience (n=8), mean (SD)	Prior rehabilitation experience (n=7), mean (SD)			No rehabilitation experience (n=8), mean (SD)	Prior rehabilitation experience (n=7), mean (SD)				
1	2.50 (0.53)	3.28 (0.75)	2.86 (0.74)	Poor –	4.25 (0.46)	4.28 (0.48)	4.27 (0.46)		Good +	
2	2.87 (0.83)	2.14 (0.69)	2.53 (0.83)	Average –	2.12 (0.64)	2.00 (1.15)	2.06 (0.88)		Good –	
3	3.25 (1.03)	3.42 (0.53)	3.33 (0.81)	Poor	4.37 (0.74)	4.00 (0.58)	4.20 (0.68)		Good	
4	3.12 (0.99)	1.85 (0.37)	2.53 (0.99)	Poor –	2.12 (0.99)	1.85 (0.69)	2.00 (0.85)		Poor –	
5	2.37 (0.51)	2.71 (1.38)	2.53 (0.99)	Poor –	3.75 (0.46)	3.71 (0.95)	3.73 (0.99)		Average +	
6	2.75 (0.70)	2.14 (0.69)	2.47 (0.74)	Poor	2.87 (0.99)	2.00 (0.82)	2.46 (1.11)		Poor	
7	3.12 (0.64)	3.57 (0.53)	3.33 (0.62)	Poor –	4.25 (0.71)	3.85 (0.89)	4.06 (0.80)		Good –	
8	2.00 (0.53)	2.42 (0.53)	2.20 (0.56)	Average	2.00 (0.75)	2.57 (0.79)	2.27 (0.80)		Average	
9	3.12 (0.83)	4.00 (0.58)	3.53 (0.83)	Poor	4.00 (0.53)	4.14 (0.69)	4.07 (0.59)		Good –	
10	3.12 (1.25)	2.42 (0.79)	2.80 (1.08)	Poor –	2.12 (0.64)	2.00 (0.58)	2.07 (0.59)		Average	

^a^SUS: System Usability Scale.

^b^TCRR: traditional card recognition rehabilitation.

^c^ISGR: interactive somatosensory game rehabilitation.

### Analysis of the Effect of Prior Rehabilitation Experience

One objective of the study was to determine the degree of acceptance of the novel ISGR among the elderly who previously went through TCRR, and to further understand whether prior rehabilitation experience would affect the willingness to use the novel rehabilitation method. Therefore, the 2 rehabilitation methods were analyzed separately for those with and without rehabilitation experience, the results of which are shown in Table 4. Table 4 revealed that regardless of prior rehabilitation experience, there were no significant differences in the answers of the elderly after TCRR or ISGR. For example, for item 1 in the SUS, “I think that I would like to use this TCRR/ISGR frequently,” the *t* value of TCRR and ISGR was –0.64 (*df*=13, *P*=.53) and –0.15 (*df*=13, *P*=.89), respectively, both of which indicated insignificant differences. This result showed that for elders, their willingness to use the ISGR was not significantly affected by prior rehabilitation experience. Hence, regardless of prior rehabilitation experience, the elderly demonstrated consistent subjective feelings of use (willingness to use) toward both TCRR and ISGR, that is, their intention to use and motivation for ISGR would not be compromised because of complicated operations. This finding was further supported by the elders’ answers to item 3, “I thought the TCRR/ISGR was easy to use.” The *t* value of TCRR and ISGR for this question was –0.41 (*df*=13, *P*=.69) and 1.08 (*df*=13, *P*=.30), respectively, again showing insignificant differences. Further analysis suggested that despite insignificant differences, the average score of ISGR (4.20) was considerably higher than that of TCRR (3.33). This result revealed the fact that the elderly believed the ISGR was easier to operate/use (please refer to the following sections for more details).

**Table 4:** Independent sample *t* test of the effect of prior rehabilitation experience.

Item	TCRR^a^			ISGR^b^	
	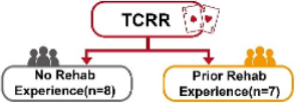			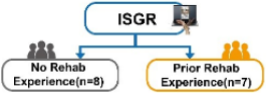	
	*t*_13_ value	*P* value	*t*_13_ value		*P* value
1	–0.64	.53	–0.15		.89
2	1.83	.09	0.26		.79
3	–0.41	.69	1.08		.30
4	2.03	.06	0.60		.56
5	–0.14	.89	0.09		.93
6	1.68	.12	1.85		.09
7	–1.45	.17	0.95		.36
8	–0.90	.38	–0.96		.36
9	–1.10	.29	–0.45		.66
10	1.27	.23	0.39		.70

^a^TCRR: traditional card recognition rehabilitation.

^b^ISGR: interactive somatosensory game rehabilitation.

### Analysis of the Effect of Gender

In this section, the 2 rehabilitation methods were analyzed separately for patients of different gender (ie, males and females) to see if the gender factor exhibited any effects on the willingness to use the TCRR or ISGR. Table 5 indicates that participants of different gender showed significant differences in the “Easy to learn” section of the ISGR. For example, for item 7, “I would imagine that most people would learn to use this TCRR/ISGR very quickly,” the *t* value of TCRR was –0.54 (*df*=13, *P*=.59, indicating insignificant difference between gender), whereas that of ISGR was 2.71 (*df*=13, *P*=.02), which showed significant differences. The results suggested that the novel ISGR developed in this study was easier to learn for females. Based on the findings, it was concluded that while the willingness to use (item 1: *t*_13_=0.98, *P*=.34, insignificant difference) and ease of use (item 3: *t*_13_=1.08, *P*=.30, insignificant difference) of the ISGR were not affected by the gender factor, this was not the case for the ease to learning (item 7). The results indicated that females were more willing to learn new things than males were; this should be investigated in future research.

**Table 5:** Independent sample *t* test of the effect of gender.

Item	TCRR^a^		ISGR^b^	
	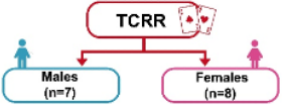		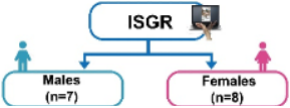	
	*t*_13_ value	*P* value	*t*_13_ value	*P* value
1	0.04	.96	0.98	.34
2	1.08	.30	0.85	.41
3	–0.41	.69	1.08	.30
4	0.90	.38	0.60	.56
5	0.90	.38	–1.42	.18
6	0.18	.86	0.65	.53
7	–0.54	.59	2.71	.02^c^
8	–0.54	.60	–0.08	.93
9	–0.77	.45	0.39	.70
10	1.27	.23	0.39	.70

^a^TCRR: traditional card recognition rehabilitation.

^b^ISGR: interactive somatosensory game rehabilitation.

^c^Significant value.

### Comparison Between TCRR and ISGR

In the previous sections, effects of “prior rehabilitation experience” and “gender” were analyzed. The results showed that prior rehabilitation experience did not affect the elderly’s willingness to use (item 1) and ease of use (item 3). However, the ease of learning (item 7) was affected by the gender factor. In this section, elders with and without rehabilitation experience were divided into separate groups to analyze their respective subjective feelings (willingness to use) of the 2 rehabilitation methods (ie, TCRR and ISGR; as shown in Table 6).

From Table 6 (column 2), significant differences between the 2 rehabilitation methods were observed on item 1 (*t*_28_=–8.27, *P*<.001), item 3 (*t*_28_=–3.17, *P*<.001), item 5, “I found the various functions in this TCRR/ISGR were well integrated” (*t*_28_=–5.07, *P*<.001), item 7 (*t*_28_=–2.81, *P*=.01), and item 10, “I needed to learn a lot of things before I could get going with this TCRR/ISGR” (*t*_28_=2.04, *P*=.03). The results suggested that the novel ISGR proposed in this study showed better “willingness to use,” “ease of use,” “integration,” “ease of learning,” and “system complexity” than TCRR. The elders indicated that they were more willing to use the novel method continuously, which was of great significance for the long-term rehabilitation of patients with dementia.

**Table 6:** Independent sample *t* test for TCRR^a^ and ISGR^b^.

Item	Comparison of the 2 rehabilitation methods (n=15)			Prior rehab experience (n=7)			No rehab experience (n=8)	
	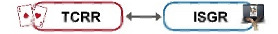			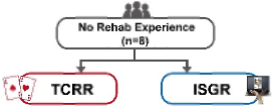			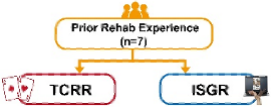	
	*t*_28_ value	*P* value	*t*_12_ value		*P* value	*t*_14_ value		*P* value
1	–8.27	<.001^c^	–4.62		<.001^c^	–7.00		<.001^c^
2	1.49	.15	0.28		.78	2.02		.06
3	–3.17	<.001^c^	–1.92		.08	–2.50		.02^c^
4	0.72	.48	0.00		.99	0.85		.41
5	–5.07	<.001^c^	–2.50		.03^c^	–5.60		<.001^c^
6	0.00	.99	0.35		.11	–0.30		.75
7	–2.81	.01^c^	–0.72		.48	–3.33		<.001^c^
8	0.39	.70	0.28		.79	0.27		.79
9	–1.21	.23	–0.42		.68	–1.18		.26
10	2.04	.03^c^	1.16		.27	2.02		.06

^a^TCRR: traditional card recognition rehabilitation.

^b^ISGR: interactive somatosensory game rehabilitation.

^c^Significant value.

Further analysis was performed by dividing participants into those with and without prior rehabilitation experience. Table 6 (columns 3 and 4) showed that for the elderly with and without prior rehabilitation experience toward TCRR and ISGR, significant differences were seen in item 1 on “willingness to use” (*t*_12_=–4.26, *P*<.001 and *t*_14_=–7.00, *P*<.001, respectively) and item 5 on “integration” (*t*_12_=–2.50, *P*=.03 and *t*_14_=–5.60, *P*<.001, respectively). This is consistent with the previous finding that “Regardless of prior rehabilitation experience, the elderly consistently showed improved willingness to use the novel ISGR over the TCRR and believed that the integration of ISGR was better.”

Comparison between the 2 rehabilitation methods suggested that for the elderly without prior rehabilitation experience (Table 6, column 4), significant differences were seen in item 3 on “ease of use” (*t*_14_=–2.50, *P*=.20) and item 7 on “ease of learning” (*t*_14_=–3.33, *P*<.001). This finding indicated that in terms of “ease of use” and “ease of learning,” the ISGR was easier for the elderly, especially for those without prior rehabilitation experience to master and operate. Thus, if an individual was exposed to the 2 rehabilitation methods for the first time, he or she would find the ISGR much easier to learn and use, which would in turn significantly affect the willingness of the elderly to continue rehabilitation. The results also echoed the conclusions of previous sections.

For item 10 on “system complexity” (I need to learn a lot...), the 2 rehabilitation methods showed significant differences (*t*_28_=2.04, *P*=.03; Table 5, column 2), but differences between the elderly with and without prior rehabilitation experience were insignificant (*t*_12_=1.16, *P*=.27 and *t*_14_=2.02, *P*=.06, respectively). This result suggested that regardless of prior rehabilitation experience, the elderly believed that it took them a decent amount of time to learn and become familiar with the TCRR before they were willing to continue rehabilitation. By contrast, the elderly could rapidly master the novel ISGR (because they could use intuitive gesture recognition during rehabilitation) and were more willing to use it in the long term.

## Discussion

### Principal Findings

In summary, elderly participants provided positive feedback on the ISGR developed in this study. The novel method would not cause the elderly to feel stressful or reluctant under the same activity and treatment arrangement of traditional methods, making it applicable for the general elderly population. Furthermore, the combination of rehabilitation with interactive games not only improved the user’s motivation for rehabilitation, but also enhanced the ease of use and learning of the system through intuitive gesture recognition technology, thereby promoting the user’s willingness to continuously engage in rehabilitation.

### Recommendations for Future Research

This study proposed the integration of a novel interactive somatosensory game into the hand rehabilitation of the elderly with MCI. We also provide suggestions on “interactive somatosensory” and “game design” for future research.

#### Interactive Somatosensory

During literature review, questionnaire survey, and experiments, it was found that patients with dementia were highly interested in interactive somatosensory games. Furthermore, the replacement of traditional rehabilitation methods with intuitive gesture recognition made it easy for the elderly to learn to use rehabilitation devices, which was consistent with findings of previous studies [5,27]. Therefore, it is recommended that subsequent research can develop various cognitive training modules based on the ISGR method developed in this study. Furthermore, a system on the medical network that allows medical institutions of different levels and even home caregivers to download interactive somatosensory games can be established so that the rehabilitation of the elderly can be performed through telemedicine or homecare [24-26].

#### Game Design

Observations during experiments indicated that using a nostalgic style in the game design was more effective in reminding the elderly of memories and prompting them to share their experience, which was consistent with findings of previous research [36,37]. Furthermore, interactive games not only demonstrate a cognitive training effect, but are also capable of increasing user stickiness by introducing various game scenarios and difficulty levels, thereby productively improving the elderly’s willingness and motivation for rehabilitation. Therefore, when designing interactive games, researchers should deeply discuss the humane, social, cultural, and other features of the elderly population and integrate them into the game content to make the elderly feel familiar with the nostalgic scenes in the game. This finding is consistent with the results of the study by Cotelli et al [38] and should be further exploited in future research.

### Conclusions

With an aging society as well as an increasing number of patients with dementia, it is of great significance to prevent and delay the incidence of dementia. Exercise can not only effectively activate the brain, but also strengthen the effect of brain activities through hand exercise as well as improving the blood circulation of the body, making it beneficial in preventing and delaying dementia. This study combined hand exercise with nostalgic therapy and integrated interactive games into the cognitive training of the elderly, so as to increase interest in rehabilitation and improve the elderly’s motivation to engage in rehabilitation in the long term. By asking the elderly to participate in both TCRR and ISGR, this study investigated the willingness of use of the elderly toward these 2 rehabilitation methods. Furthermore, effects of gender and prior rehabilitation experience on these 2 rehabilitation methods were explored to understand the elderly’s thoughts and feelings of ISGR. Results showed that the ease of learning of the 2 methods was considerably different. For elderly people without prior rehabilitation experience, the ISGR was easier to learn and operate than the TCRR, and females performed better in learning the novel method. The results suggested that the novel ISGR developed in this study could not only improve the elderly’s motivation for rehabilitation, but also promote them to continuously participate in rehabilitation. Therefore, it can be widely applied to other rehabilitation fields (such as home rehabilitation or telemedicine) in the future.

## Abbreviations

ISGR: interactive somatosensory game rehabilitation
LM: leap motion
MCI: mild cognitive impairment
SUS: System Usability Scale
TCRR: traditional card recognition rehabilitation
WHO: World Health Organization
